# Drug therapy management in patients with renal impairment: how to use creatinine-based formulas in clinical practice

**DOI:** 10.1007/s00228-016-2113-2

**Published:** 2016-08-27

**Authors:** Willemijn L. Eppenga, Cornelis Kramers, Hieronymus J. Derijks, Michel Wensing, Jack F.M. Wetzels, Peter A.G.M. De Smet

**Affiliations:** 1 Radboud Institute for Health Sciences, IQ healthcare, Radboud University Medical Center, Nijmegen, The Netherlands; 2 Departments of General Internal Medicine and Pharmacology and Toxicology, Radboud University Medical Center, Nijmegen, The Netherlands; 3 Department of Clinical Pharmacy, Canisius Wilhelmina Hospital, Nijmegen, The Netherlands; 4 Hospital Pharmacy ‘ZANOB’, ‘s-Hertogenbosch, The Netherlands; 5 Division of Pharmacoepidemiology and Pharmacotherapy, Utrecht Institute for Pharmaceutical Sciences (UIPS), Utrecht University, Utrecht, The Netherlands; 6 Department of Nephrology, Radboud University Medical Center, Nijmegen, The Netherlands; 7 Department of Pharmacy, Radboud University Medical Center, Nijmegen, The Netherlands

**Keywords:** Glomerular filtration rate, Renal function, Drug dose, Therapeutic window, Adverse drug reaction

## Abstract

**Purpose:**

The use of estimated glomerular filtration rate (eGFR) in daily clinical practice.

**Methods:**

eGFR is a key component in drug therapy management (DTM) in patients with renal impairment. eGFR is routinely reported by laboratories whenever a serum creatinine testing is ordered. In this paper, we will discuss how to use eGFR knowing the limitations of serum creatinine-based formulas.

**Results:**

Before starting a renally excreted drug, an equally effective drug which can be used more safely in patients with renal impairment should be considered. If a renally excreted drug is needed, the reliability of the eGFR should be assessed and when needed, a 24-h urine creatinine clearance collection should be performed. After achieving the best approximation of the true GFR, we suggest a gradual drug dose adaptation according to the renal function. A different approach for drugs with a narrow therapeutic window (NTW) is recommended compared to drugs with a broad therapeutic window. For practical purposes, a therapeutic window of 5 or less was defined as a NTW and a list of NTW drugs is presented. Considerations about the drug dose may be different at the start of the therapy or during the therapy and depending on the indication. Monitoring effectiveness and adverse drug reactions are important, especially for NTW drugs. Dose adjustment should be based on an ongoing assessment of clinical status and risk versus the benefit of the used regimen.

**Conclusion:**

When determining the most appropriate dosing regimen serum creatinine-based formulas should never be used naively but always in combination with clinical and pharmacological assessment of the individual patient.

**Electronic supplementary material:**

The online version of this article (doi:10.1007/s00228-016-2113-2) contains supplementary material, which is available to authorized users.

## Introduction

Ten to 32 % (in elderly patients) of adverse drug reactions (ADRs) that necessitated hospital admission were related to impaired renal function [1, 2]. Drug therapy adjustment according to renal function is therefore of major importance to improve drug therapy management (DTM).

The glomerular filtration rate (GFR) is widely accepted as the preferred index of the kidney function [3]. Since the introduction of the Modification of Diet in Renal Disease (MDRD) formula [4], laboratories routinely report the estimated GFR (eGFR) when serum creatinine testing is ordered [5–7]. More recently, the Chronic Kidney Disease Epidemiology Collaboration (CKD-EPI) formulas for eGFR were developed [8]. All formulas have in common are that they are based on serum creatinine levels. However, there are several factors which may influence serum creatinine levels and therefore, eGFR without affecting true GFR itself. These factors vary from analytical variations to patient characteristics, such as muscle mass and liver function [9–12]. Inaccuracy in eGFR values might lead either to overestimation of kidney function, leading to administration of inappropriately large doses and therefore possible toxicity, or, conversely, underestimation of kidney function, leading to subtherapeutic dosing and therefore treatment failure and prolonged illness [13]. The theoretical effect of the possible overestimation and underestimation of true GFR is illustrated in Online Resource 1. The inaccuracy of the eGFR may lead to a different renal function group than the renal function group to which the patient actually belongs to according the mGFR.

Despite the limitations, serum creatinine-based formulas are routinely used in daily clinical practice [14–17]. In this paper, we will discuss approaches on how to use serum creatinine-based formulas in daily clinical practice in a well-informed way.

### Risk-benefit ratio

The first question after defining the patient’s health problem is: “What is the therapeutic objective?” [18]. In general, the goal of DTM is to optimize the risk-benefit ratio and to attain an optimal therapeutic outcome [19]. In the situation of patients with renal impairment, this goal is not any different. In a lifesaving situation, for example, a treatment with antibiotics in a high dose in case of sepsis, the risk of developing ADRs is less important than the risk to give an insufficient drug dose which may lead to untimely death. In less urgent situations, such as treating hypertension, a more conservative drug dose can be given at the start of the therapy. And can be gradually increased with monitoring effect (blood pressure) and/or ADRs. In other words: “start low, go slow.”

For patients with renal impairment the risk-benefit ratio should be taken into account when answering the following questions:

1. Can I use the eGFR reported by laboratories in DTM?
2. Is there an equally effective drug which can be used more safely in patients with renal impairment?
3. What are the chances and risks of reaching drug levels outside the therapeutic window?
  - Considerations at start of pharmacotherapy
  - Considerations during pharmacotherapy
4. Is it possible to monitor effectiveness and/or ADRs in order to timely intervene and/or to prevent serious situations?

### How to apply eGFR in daily clinical practice

When there are no reasons to suspect that the true GFR is substantially different from the eGFR, it can be used without restrictions.

In cases of rapidly changing GFR, the serum creatinine levels will not reflect the actual GFR until steady-state has been reached [19, 20]. In such situations, assessment of impaired renal function must rely on multiple measurements [21]. The modified Jelliffe formula may be useful in measuring an unstable renal function because it uses pairs of serum creatinine samples instead of only one sample [22, 23]. However, further comparative studies of GFR estimation in patients with unstable renal function are still needed. In addition, it is unclear how to adjust drug doses in unstable situations.

In specific patients and/or clinical situations (e.g., malnourishment, relatively low or high muscle mass, acute critical illness) where estimating equations are known to be inaccurate or clinical decision-making requires a greater accuracy than expected from eGFR, the GFR should be measured [19, 24]. Measurement of GFR is ideally performed with gold standards such as ^51^chromium ethylenediaminetetraacetic acid (^51^Cr-EDTA), technetium-labeled diethylene-triamine-pentacetate (^99m^Tc-DTPA), iohexol, inulin, and iothalamate [10, 25]. However, these markers are impractical for routine clinical use due to limited access to necessary diagnostic facilities and high cost [26]. Twenty-four-hour urine creatinine clearance collection is easier to perform, especially in the hospital care setting, and might be helpful. It can be used to find out if the estimation is inadequate due to abnormal muscle mass. If the creatinine clearance points in the same direction as the eGFR, the eGFR can be used for decision-making. In the ambulatory care setting, this method might be more inconvenient for the patient and prone to failure of collection of the entire specimen [26, 27].

Recently, the European Medicines Agency advised to use the absolute eGFR in drug dosing [28]. However, the reason that the normalized eGFR is used worldwide is the lack of the availability of weight as a parameter. The difference between normalized (ml/min/1.73 m^2^) and absolute (ml/min) eGFR values should only be taken into account when someone is substantially larger or smaller than an average person, but with a normal figure (with a body surface area (BSA) of 1.73 m^2^). The best descriptor of body size in obese patients is still unclear [29]. The GFR increases with body size but does not increase in proportion to the total body weight [6]. Therefore, adjustment of eGFR to absolute GFR using BSA calculated with actual body weight causes errors in obese patients [6, 30]. A recent study suggests the use of ideal body weight as the body size descriptor for GFR indexation [31], although others suggest the lean body weight [30, 32].

In summary, the reported eGFR by laboratories may not reflect the true GFR of the individual patient. To make it even more complicated, most drug dose recommendations in patients with renal impairment are based on the Cockcroft and Gault (CG) formula, representing renal clearance of creatinine instead of eGFR. This might lead to clinically relevant problems, for example, when using the new thrombin inhibitors, such as dabigatran and rivaroxaban [33–35]. The use of the MDRD-4 formula instead of the CG formula (used in clinical trials) would result in higher doses or incorrect judgment if patients are eligible for treatment [34, 35]. Dabigatran would be recommended in a full dose for 33 % of all participants when using the CG formula compared to 67 % when using the MDRD-4 formula [34]. Safety has not been established using the MDRD equation. This is a concern since the risk of major bleeding or the development of thrombosis would be increased in patients with renal impairment [35].

All in all, there are many uncertainties when using eGFR in DTM. In the following paragraphs, we will outline possible considerations when prescribing drugs in patients with renal impairment and how to cope with the knowledge and uncertainties we know today.

### Choice of drug

As in every step of prescribing drugs, the benefits should outweigh the risk. In patients with renal impairment, alternatives for drugs that are renally excreted, should be considered. There are often alternatives available. For some drug classes, there are several alternatives, for example, in the drug class of statins. Only rosuvastatin is contraindicated in severe renal impairment, whereas the other statins are not [36]. If a 65-year-old woman is diagnosed with diabetes mellitus type 2 with an eGFR of 35 ml/min/1.73 m^2^ is metformin still the drug of choice? Metformin according to the guidelines may be started in a low dose of 500 mg two times daily. The risk of metformin-associated lactic acidosis increases when renal function drops below 30 ml/min/1.73 m^2^ [37]. This risk is probably already increased in the 65-year-old woman, because the eGFR is near the cutoff value of 30 ml/min/1.73 m^2^. Furthermore, the eGFR may overestimate true GFR and the renal function may decline in the near future. Gliclazide might be a better choice.

If a nephrotoxic drug (renally excreted or not) is considered to be prescribed, a more conservative approach both in prescribing the drug or choosing the drug dose might be preferable.

In some clinical situations, a renally excreted drug may be necessary. Then, the drug dose becomes important, which will be discussed in the next paragraph.

### Therapeutic window

The therapeutic window (TW) reflects the concentration range that provides efficacy without unacceptable toxicity. In other words, the area between the minimum efficacious dose and the maximum tolerable dose [38–40]. The TW may also be thought of as a range of acceptable plasma levels of the drug and its active metabolite(s) in which positive therapeutic results are seen [39]:

$$ \mathrm{Therapeutic}\ \mathrm{window}\ (TW)=\frac{\mathrm{Minimum}\ \mathrm{toxic}\ \mathrm{plasma}\ \mathrm{concentration}}{\mathrm{Minimum}\ \mathrm{effective}\ \mathrm{plasma}\ \mathrm{concentration}} $$

In order to explain the effect of the TW on reaching toxic levels, we first illustrate the effect of overestimation and underestimation of the true GFR on the relative steady-state drug level (rC_ss_) in Table 1. We assumed that an rC_ss_ of 100 % is reached in patients with a normal renal function of 100 ml/min/1.73 m^2^ and the recommended drug dose is 100 mg/day. From our clinical experience, the overestimation of the eGFR, calculated with the MDRD formula, may become as high as 50–100 % in patients who are completely bedridden for a prolonged period [41].

**Table 1:** Theoretical effects of substantial overestimation of eGFR values on relative steady-state drug levels of renally cleared drugs

eGFR (1.73 ml/min/m^2^)	Daily dose^a^ (mg)	Overestimation							
		0 %		25 %		50 %		100 %	
		Corrected eGFR	Drug level^b^ (%)	Corrected eGFR	Drug level^b^ (%)	Corrected eGFR	Drug level^b^ (%)	Corrected eGFR	Drug level^b^ (%)
100	100	100	100	80	125	67	149	50	200
60	100	60	167	48	208	40	250	30	333
40	50	40	125	32	156	27	185	20	250
20	25	20	125	16	156	13	154	10	250

^a^Recommended dose regimen: 100 mg if eGFR is >50 ml/min/1.73 m^2^; 50 mg if eGFR is 30–50 ml/min/1.73 m^2^; 25 mg if eGFR is 10–30 ml/min/1.73 m^2^

^b^Relative steady-state drug level (rCss) has been calculated as follows: $\mathrm{Relative}\ \mathrm{steady}\ \mathrm{state}\ \mathrm{drug}\ \mathrm{level}=\frac{\mathrm{corresponding}\ \mathrm{drug}\ \mathrm{dose}\ \mathrm{recommendation}\ \left(\mathrm{mg}/\mathrm{day}\right)}{\mathrm{eGFR}\ \mathrm{corrected}\ for\ \mathrm{overestimation}}$

This formula is a simplification of the formula [55]

$Css=\frac{\mathrm{Bioavailability}\times \mathrm{drug}\ \mathrm{dose}}{\mathrm{Dosing}\ \mathrm{interval}\times \mathrm{drug}\ \mathrm{clearance}}$ by making the following assumptions[55]:

-The patient has a normal body surface area of 1.73 m^2^

-The drug has a bioavailability of 1 (i.e., 100 %)

-The drug has a dosing interval of 1 (i.e., once daily)

-The drug is completely renally cleared

Theoretically, when the TW of a drug is 2, with a minimum relative effective plasma concentration of 70 % and a minimum relative toxic plasma concentration of 140 %, patients may suffer from a toxic rC_ss_ (see Table 1). For example, when the GFR is overestimated by 25 %, patients suffering from impaired renal function (<60 ml/min/1.73 m^2^) will reach an rC_ss_ that exceeds 140 %. If the TW is assumed to be 3 corresponding to a relative therapeutic range of 70–210 %, toxic levels could also emerge easily. However, if the TW rises to 10 (70–700 %, respectively), it becomes much more difficult to reach toxic levels (unless the drug level is already near the minimum toxic level).

In the sequel of the definition of the TW, the question arises: when is a TW called a narrow therapeutic window (NTW)?

Recently, Schulz et al. reported for nearly 1000 drugs and other xenobiotics, therapeutic (“normal”), and, if data were available, toxic and comatose/fatal blood plasma concentrations [42]. The ranges reported for therapeutic and toxic blood plasma concentrations could be transformed to the presented formula earlier.

Definitions for NTW drugs are lacking in the literature. We suggest that a drug with a TW of 5 or lower can be arbitrarily defined as a drug with a NTW. Online Resource 2 presents examples of renally excreted drugs (or their active metabolites) with a TW of 5 or less. We used the Dutch guidelines for drug dosing in chronic kidney disease to select drugs which need dose adjustment, are contraindicated, or need therapeutic drug monitoring in renal impairment [36]. The TW was calculated with the information summarized by Schulz et al. [42] and supplemented with recent literature which indicates that toxic levels may be reached easily in patients with renal impairment [36, 42]. It appeared that for many drugs, we could not retrieve concrete TW data. Therefore, this list should be considered as a starting point, which has to be updated when new information comes available. We recommend a different approach for drugs with an NTW than for drugs with a broad therapeutic window (BTW) for drug dosing in patients with renal impairment [19].

Of note, clinicians should also be aware when a high dose (near the maximum recommended dose) is needed of a drug without an NTW, for example, amoxicillin. In the situation of bacterial meningitis, for example, patients with renal impairment are at increased risk to reach plasma concentration above the minimum toxic plasma concentration, because the minimum effective dose and therefore the plasma level is higher. A lower dose should be considered in those situations [43].

#### Considerations at the start of pharmacotherapy

When a renally cleared drug is needed for a patient with renal impairment, the starting dose should be considered. The rate at which the effect of the drug must be achieved (quickly or not) is of major importance. The following questions should be asked: (1) what is the risk of therapeutic failure with lower doses, and (2) what is the risk of drug toxicity with higher doses [43]?

If the pharmacological effect is needed quickly, one should consider starting with the recommended dose for patients with normal renal function. This might be seen as a loading dose and the dose might be adjusted depending on ADRs and/or effectiveness. An example is antibiotics. The risk when dosing too low is insufficient efficacy, but also increasing risk to develop drug resistance. For most antibiotics, the ADRs are easy to observe and may be relatively mild. Therefore, starting with a normal dose is preferable.

In case it is not crucial to have a quick pharmacological effect, one should preferably use the “start low and go slow principle”. Examples are statins and antihypertensive drugs. Especially, for drugs with an NTW, one should consider a more conservative approach [44].

#### Considerations during pharmacotherapy

Drug dose recommendations concerning patients with impaired renal function are usually expressed per renal function category (50–80, 30–49, 10–29, and <10 ml/min/1.73 m^2^) [36, 45]. Therefore, recommended dose changes for most drugs are crude (e.g., halving the dose or changing from twice-a-day regimen to a once-a-day regimen) [14]. One could argue that differences between eGFR and the true GFR will remain without practical consequences as long as they do not result in different renal function categories. However, the factors influencing the variance of the eGFR become more important as the eGFR approaches the nearest cutoff value for falling into another renal function category. Then, a minor change in eGFR over time will lead to different drug dosing recommendations. It is important to keep in mind that different renal function categories to guide drug dosing are merely a derivative of a continuous function that is expressed by the following formula:

$$ \mathrm{fraction}\ \mathrm{of}\ \mathrm{normal}\ \mathrm{dose}=1\hbox{--} {f}_e\times \left(1\hbox{--} {k}_F\right) $$

herein, *f*_*e*_ is the fraction of the original dose excreted as unchanged compound (or active metabolite) in the urine, while *k*_*F*_ is the patient’s GFR divided by 120 mL/min [46]. If one indiscriminately applies drug dose recommendations of 50 % for eGFRs of 50–30 ml/min and of 25 % for eGFRs of 30–10 ml/min, a minor change in eGFR from, for example, 31 to 29 ml/min will halve the drug dose. If it is assumed that the *f*_*e*_ for the particular drug equals 1, the formula earlier will yield a dose of 26 and 24 % for eGFRs of 31 and 29 ml/min, respectively. Alternatively, one could decide to replace the recommended doses of half and a quarter of the full drug dose by one third for eGFRs around 30 ml/min [7]. Following the calculation of the desired drug dose, the prescribed drug dose must be rounded off to the available strengths of the drug in question [47].

### Monitoring

Drug efficacy and safety should be monitored after the start of the therapy. Drug therapy management should be based on ongoing assessment of clinical status and risk versus benefit of the current regimen [19]. The Dutch guideline for drug dosing in patients with renal impairment generally recommends therapeutic drug monitoring (TDM) and/or careful monitoring of therapeutic effects and/or ADRs for all drugs with a narrow therapeutic window (see Online Resource 2) [36]. When it is not possible to monitor effectiveness or ADRs appropriately, or when it is not possible to timely detect serious ADRs, use of a different drug should be considered.

For a number of drugs monitoring their effectiveness and/or ADRs (when feasible through TDM) is more important than dose adjustment according to current guidelines. Digoxin, for example, is a drug that is difficult to manage, particularly in elderly patients who are at high risk of decreased renal function [48]. High inter-individual variation for digoxine plasma levels have been observed [49, 50]. Therefore, therapeutic drug monitoring of digoxin is indicated. Another example is allopurinol. Treat to target serum uric acid concentrations (<0.36 mmol/l) rather than give a dose according to renal function has been shown to be safe and effective [51, 52]. The potential benefits of TDM in patients with renal impairment has also been observed for ciprofloxacin and amoxicillin/clavulanic acid. In recent studies, the standard doses frequently resulted in underdosing, especially in younger patients with good creatinine clearance [53, 54]. One cannot always rely on recommended doses.

## Conclusion

DTM based naively on eGFR is not without limitations. In addition, multimorbidity and drug-drug interactions can further complicate clinical decision-making. [42]. The narrower the therapeutic window of a drug is, the more relevant individual patient characteristics are and the less satisfactory crude dose recommendations become [14].

Therefore, the following considerations in DTM in patients with renal impairment should be made: (1) Is the drug renally excreted? If yes, is there a safer alternative available?; (2) If not, is it possible that the eGFR substantially deviates from the true GFR? If yes, consider 24-h urine creatinine clearance collection; (3) Does the BSA of the patient deviates substantially from 1.73 m^2^? If yes, calculate BSA and adjust eGFR to milliliters per minute; (4) Combine consideration 2 and 3 in order to achieve the best approximation of the true GFR; (5) Adjust the drug dose gradually to the renal function; (6) Be extra careful with drugs with an NTW and consider another starting dose depending on indication; (7) Consider if it is possible to monitor effectiveness and/or ADRs with TDM and/or other measurements.

In conclusion, when determining most appropriate dosing regimen for patient with impaired renal function, the serum creatinine-based formulas should never be used naively but always in combination with clinical and pharmacological assessment of the individual patient [44].

### Author Contributions

Eppenga, Kramers, Derijks, Wensing, Wetzels, and de Smet drafted and revised the manuscript critically. Eppenga, Kramers, Derijks, Wetzels, Wensing, and de Smet worked on the final approval of the manuscript.

## Electronic supplementary material

Online Resource 1 (PDF 56.8 kb)

Online Resource 2 (PDF 106 kb)
