# Supplementary material for: Drug therapy management in patients with renal impairment: how to use creatinine-based formulas in clinical practice
Source: Eur J Clin Pharmacol. 2016 Aug 27;72(12):1433–9. doi: 10.1007/s00228-016-2113-2 (PMC5110609; doi:10.1007/s00228-016-2113-2)
Supplement: Supplementary file 1 — (PDF 56.8 kb) [file 228_2016_2113_MOESM1_ESM.pdf]

Online Resource 1 The effects of the inaccuracy of the eGFR in drug dosing

		**Renal function groups for drug dosing[44]**				
**mGFR (ml/min/1.73m^2)**	**eGFR (mGFR ± 30%)***	**< 10**	**10-30**	**30-50**	**50-80**	**> 80**
100	70-130					
60	42-78					
40	28-52					
20	14-26					

eGFR = estimated glomerular filtration rate; mGFR = measured glomerular filtration rate

* An accuracy expressed as $P_{30\%}$ (eGFR falls within ± 30% of the mGFR) of 80% or higher has been indicated as sufficient.[24, 56, 57]

The grey parts in the table illustrate the effects of the inaccuracy of the eGFR (mGFR ± 30%). The eGFR may lead to a different renal function group than the renal function group to which the patient actually belongs according to the mGFR.
