# Supplementary material for: Drug therapy management in patients with renal impairment: how to use creatinine-based formulas in clinical practice
Source: Eur J Clin Pharmacol. 2016 Aug 27;72(12):1433–9. doi: 10.1007/s00228-016-2113-2 (PMC5110609; doi:10.1007/s00228-016-2113-2)
Supplement: Supplementary file 2 — (PDF 106 kb) [file 228_2016_2113_MOESM2_ESM.pdf]

**Online Resource 2 Renally cleared drugs with a narrow therapeutic window.**

| | **Type of advice in patients with renal impairment** | | | | |
|---|---|---|---|---|---|
| **Drug** | **Dose adaptation** | **Therapeutic drug monitoring** | **Monitoring therapeutic effect (TE) and/or adverse drug reactions (ADR)** | **Therapeutic window based on Schulz et al.[42]** | **Other references** |
| *Analgetic and antirheumatic drugs* | | | | | |
| Hydroxychloroquine | | | √ (ADR) | 5 | |
| *Antibacterial drugs* | | | | | |
| Amikacin | √ | √ | | 3 | [38, 58] |
| Ciprofloxacin | √ | | √ (ADR) | 4.6 | |
| Gentamicin | | √ | | 3 | [38, 58] |
| Tobramicin | | √ | | 3 | [38] |
| *Antiepileptic drugs* | | | | | |
| Carbamazepine | | √ | √ (ADR) | 5 | [38, 59] |
| Oxcarbazepine | √ | | √ (TE) | 4.5 | |
| Pregabaline | √ | | √ (ADR) | 5 | |

| | | | | | |
|---|---|---|---|---|---|
| Primidone | | | √ (TE, ADR) | 5 | [57] |
| Zonisamide | √ | | √ (ADR) | 4 | |
| *Antiglaucoma drugs* | | | | | |
| Acetazolamide | √ | | √ (ADR) | 2.5 | |
| *Antigout drugs* | | | | | |
| Allopurinol | √ | | √ (ADR) | 4[¶] | |
| *Antimycotica* | | | | | |
| Flucytosine | √ | √ | | 2.86 | |
| Voriconazol intravenous | √[*] | | | 1.75 | |
| *Antiparkinsonian drugs* | | | | | |
| Amantadine | √ | | √ (ADR) | 5 | |
| *Antipsychotic drugs* | | | | | |
| Lithium | √ | √ | | 3.25 | [38, 58, 59] |
| *Cardiac drugs* | | | | | |
| Digoxin | √ | √ | √ (TE, ADR) | 5 | [38, 58, 59] |
| Disopyramide | | √ | √ (TE) | 4 | |
| Flecainide | | √ | √ (TE) | 2.5-5 | [38] |
| Milrinon | √ | | √ (ADR) | 2 | |
| *Gastrointestinal drugs* | | | | | |

| | | | | | |
|---|---|---|---|---|---|
| Metoclopramide | √ | | √ (ADR) | 4 | |
| *Various* | | | | | |
| Memantine | √ | | √ (ADR) | 3.3 | |
| Varenicline | √ | | √ (ADR) | 2.5 | |

[*] Voriconazol by the intravenous route is contraindicated in patients with moderate to severe renal impairment. This is due to the potential toxic effects of the accumulation of the solvent vehicle sulphobutyletherbetacyclodextrin.[60]

[¶] The therapeutic window is based on the information of the metabolite oxypurinol.
